# To *g* or Not to *g*? A Cross-Domain, Subtest-Level Investigation of the Flynn Effect Across Ages 7–15 Years

**DOI:** 10.1080/00273171.2026.2707653

**Published:** 2026-08-05

**Authors:** Evan J. Giangrande, Sean R. Womack, Deborah Finkel, Deborah W. Davis, Christopher R. Beam, Eric Turkheimer

**Affiliations:** a Analytic and Translational Genetics Unit and Psychiatric and Neurodevelopmental Genetics Unit, Massachusetts General Hospital, Boston, MA, USA; b Stanley Center for Psychiatric Research, Broad Institute of MIT and Harvard, Cambridge, MA, USA; c Department of Psychology, University of Virginia, Charlottesville, VA, USA; d Center for Economic and Social Research, University of Southern California, Los Angeles, CA, USA; e Institute for Gerontology, Jönköping University, Jönköping, Sweden; f Department of Pediatrics, University of Louisville School of Medicine, Louisville, KY, USA; g Department of Psychology, University of Southern California, Los Angeles, CA, USA

**Keywords:** Flynn Effect, cognitive ability, longitudinal, MIMIC, intelligence

## Abstract

For decades, researchers have debated whether the magnitude of the Flynn Effect—intergenerational increases in mean IQ scores—varies across cognitive domains and subdomains, and whether the Flynn Effect reflects gains in general cognitive ability (*g*). We conducted a cross-domain, subtest-level investigation of the Flynn Effect across middle childhood and early adolescence (ages 7–15 years, *N* = 1187, 89% White, 9% Black, 52% female) in longitudinal cognitive ability data collected prospectively between 1957 and 1999 using three versions of the Wechsler Intelligence Scale for Children. Results provided clear evidence of the Flynn Effect as both increases in mean IQ score across generational cohort and decreases in mean IQ across test versions. Flynn Effect magnitude differed substantially across domains and subtests. Performance IQ gains were larger than full-scale and verbal IQ gains in test version-based analyses, but cohort-based estimates showed modest differences in an opposite pattern (VIQ > FSIQ > PIQ). Variance in Flynn Effect magnitude across subtests did not follow a discernible pattern. The strength of the Flynn Effect on individual subtests was not strictly proportional to each subtest’s *g*-loading, with performance IQ subtests showing larger increases than would be expected given their *g*-loadings and verbal subtests showing smaller-than-expected gains.

Mean IQ scores have risen dramatically across generations in socioeconomically developed countries. James Flynn (Flynn, 1984, 1987) first documented secular increases in mean IQ, now known as the Flynn Effect, and both his work and meta-analytic findings suggest that scores rose approximately 3 points per decade over at least the twentieth century (Pietschnig & Voracek, 2015; Trahan et al., 2014; Wongupparaj et al., 2023). The Flynn Effect may be even larger in developing countries (Daley et al., 2003) and may asymptote in developed countries (Pietschnig & Voracek, 2015; Wongupparaj et al., 2015), suggesting that IQ gains are linked to environmental enrichment at a societal level. Indeed, the Flynn Effect highlights the importance of environmental influences on cognitive development, as intergenerational genetic changes alone are too small to explain its magnitude (Bratsberg & Rogeberg, 2018); the effect likely arises from a complex interplay of genetic and environmental factors (Dickens & Flynn, 2001; Giangrande et al., 2022; Pietschnig & Voracek, 2015). Researchers have debated whether the effect has continued, slowed, or even reversed in recent years (Dworak et al., 2023; Rodgers, 2023; Teasdale & Owen, 2005). Unmodeled Flynn Effects can massively confound both cross-sectional (Dickinson & Hiscock, 2010) and longitudinal analyses (O’Keefe & Rodgers, 2020) that include multiple generational cohorts or multiple cognitive ability test versions. The Flynn Effect also has profound practical and clinical implications (Flynn & Widaman, 2008), particularly in situations where IQ cutoffs are used including intellectual disability diagnosis (Kanaya, Ceci, et al., 2003; Kanaya, Scullin, et al., 2003; Scullin, 2006), learning disability diagnosis (Kanaya & Ceci, 2012), and eligibility for the death penalty (Flynn, 2006).

Since Flynn’s (1984, 1987) original studies, intergenerational gains in cognitive ability have been observed repeatedly in hundreds of samples from around the world, using a variety of methods (Pietschnig & Voracek, 2015; Trahan et al., 2014; Wongupparaj et al., 2023). Most frequently, studies have documented the Flynn Effect using one of two approaches, which Flynn (1984, 1987) himself also used. The first approach (e.g., Flynn, 1984) involves giving the same sample two different versions of an IQ test, an older and a newer version. Because IQ tests are periodically re-standardized to a mean of 100, observing substantially lower mean IQ scores on the newer test version than the older version indicates that IQ scores rose over time until the test was re-standardized back down to a mean of 100. In addition to these *test version effects*, the Flynn Effect can also be documented by giving the same cognitive ability test to multiple generational cohorts and noting that more recent cohorts scored higher on average than earlier cohorts (*cohort effects*; e.g., Flynn, 1987). The test version and cohort approaches both have limitations. Test version effects can be influenced by measurement artifacts (Beaujean & Osterlind, 2008; Ceci & Kanaya, 2010; Gonthier & Grégoire, 2022; Kaufman, 2010; Wicherts et al., 2004). Cohort effects, particularly in studies where data have been collected over a long time span, are vulnerable to confounding by period effects (O’Keefe et al., 2023) and changes in data collection (e.g., sample selection). One previous study observed both test version and cohort effects simultaneously in the same sample, increasing confidence in the Flynn Effect’s robustness (Giangrande et al., 2022).

## What is rising?

Despite decades of research and extensive replication, scholars continue to debate what exactly the Flynn Effect tells us about human cognitive ability. Measures of *fluid intelligence* (problem solving and abstract reasoning that does not rely on previous knowledge; measures include performance IQ) tend to show greater gains than measures of *crystallized intelligence* (application of knowledge previously acquired through experience and education; measures include verbal IQ) (Pietschnig & Voracek, 2015). To the extent that gains vary across cognitive domains and subdomains, this has ramifications for both research and practice (e.g., how to correct for the Flynn Effect in cognitive development studies and update IQ cutoffs in diagnostic decision-making) (Kanaya & Ceci, 2011). In light of observed differential Flynn Effects across cognitive domains, some have questioned whether it is appropriate to conclude that mean *intelligence* has risen over time (e.g., Jensen, 1998). Specifically, some have argued that if the Flynn Effect arises from (sub)-domain-specific gains rather than “genuine” increases in general cognitive ability, the Flynn Effect is “hollow;” although people have gotten better at certain subtests, intelligence per se has not increased across generations (Jensen, 1998). Others have argued that differential gains are to be expected: intergenerational improvements in environmental conditions have facilitated cognitive specialization, which leads to variability in Flynn Effect size across (sub)domains that may not be directly proportional to gains in general cognitive ability (Flynn et al., 2014; Oberleiter et al., 2024).

Several previous studies have attempted to address these questions by examining whether the Flynn Effect reflects intergenerational gains in psychometric *g*, a latent factor commonly used to quantify general intelligence (Andrzejewski et al., 2024; Colom et al., 2001, 2023; Lazaridis et al., 2022; Must et al., 2003; Oberleiter et al., 2024, 2025; Pietschnig et al., 2023; Pietschnig & Voracek, 2015; Te Nijenhuis, 2013; Wicherts et al., 2004). Some studies (e.g., Colom, Juan-Espinosa, & García, 2001) have tested whether gains on subtests are proportional to subtests’ *g* loadings. Others (e.g., Andrzejewski et al., 2024; Oberleiter et al., 2024, 2025) have tracked the strength of intercorrelations among subtests, known as the *positive manifold* of intelligence, over time. These studies are based on the supposition that observing subtest Flynn Effects together with cross-temporal stability in the positive manifold would suggest that *g* has risen, while decreasing intercorrelation strength is interpreted as evidence that subtests have increased independently of *g*. However, such patterns do not map directly onto changes in the mean of *g* because the magnitude of intercorrelations also depends on factor loadings, residual variances, and other features of the measurement model (Wicherts et al., 2004). Results have been contradictory, with some studies finding that subtest gains are consistent with *g* gains (Colom et al., 2001; Oberleiter et al., 2025) and others concluding that subtest-specific gains are not fully related to *g* gains (Andrzejewski et al., 2024; Must et al., 2003; Pietschnig & Voracek, 2015; Te Nijenhuis, 2013).

This inconsistency may stem in part from several shared limitations. First, of the hundreds of Flynn Effect studies that have been conducted to date, relatively few have examined whether (sub)domain-level gains are associated with *g* gains (Andrzejewski et al., 2024; Colom et al., 2001, 2023; Lazaridis et al., 2022; Must et al., 2003; Oberleiter et al., 2024, 2025; Pietschnig et al., 2023; Pietschnig & Voracek, 2015; Te Nijenhuis, 2013; Wicherts et al., 2004). This rarity likely stems from a lack of appropriate data sets; relatively few studies have collected data across multiple subtests and multiple cohorts or test versions. Additional work is needed to clarify the Flynn Effect’s relative magnitude across cognitive domains and subdomains and the extent to which such gains are associated with *g*. Second, all but one previous Flynn Effect study (Giangrande et al., 2022) have examined either test version or cohort effects in isolation, and no previous domain or subdomain-level analysis of which we are aware has examined test version and cohort effects simultaneously. Previous results are therefore vulnerable to the respective limitations of each approach. Third, nearly all previous Flynn Effect studies—especially those focused on *g*—have been cross-sectional. Longitudinal models provide more stable estimates of differences among cohorts (Schaie et al., 2005) and are more statistically powerful than cross-sectional models (Giangrande et al., 2019). They also make it possible to differentiate cohort effects from aging-related cognitive ability differences (Dickinson & Hiscock, 2010). Several studies have fit longitudinal Flynn Effect models without focusing on *g* (Giangrande et al., 2022; Kanaya et al., 2005; Karlsson et al., 2015; O’Keefe & Rodgers, 2017, 2019, 2020; Skirbekk et al., 2013; Thorvaldsson et al., 2017; Wänström et al., 2023). Rodgers & Wänström (2007) and Rodgers et al. (2025) performed cross-temporal analyses in data from the National Longitudinal Survey of Youth. Flynn Effect experts have called for additional longitudinal work to fill these gaps, including latent growth curve approaches (Kanaya et al., 2005; Rodgers, 2023; Wänström et al., 2023).

## Current study

In this study, we examined the extent to which the Flynn Effect varies in magnitude across cognitive domains and subtests, and whether observed gains on a given subtest were proportional to *g* loadings. We developed novel models to address previous limitations in Flynn Effect research while providing a more nuanced understanding of intergenerational changes in cognitive ability. Data were drawn from the Louisville Twin Study (LTS), a comprehensive, longitudinal study of cognitive development. Flynn Effects on cognitive ability index scores (i.e., IQ), both traditionally estimated group-level effects and within-person effects that boost individual-level cognitive growth, have been previously documented in the LTS sample across age 7–15 years (Giangrande et al., 2022). The present study used data from the same age range. Although the LTS is a twin study, we did not conduct genetically informed analyses here; Giangrande and colleagues (Giangrande et al., 2022) explored genetic and environmental influences on individual sensitivity to the Flynn Effect.

Several aspects of the LTS make it uniquely suited to Flynn Effect analyses. First, and most importantly for the present study, measures of both general intelligence and domain-specific cognitive functioning are available. WISC subtest scores were collected for all participants, making it possible to estimate Flynn Effects on specific cognitive domains (i.e., subtests), crystallized and fluid intelligence (measured by verbal IQ and performance IQ, respectively), and general cognitive ability (full-scale IQ). This sets the stage for cross-domain comparisons of Flynn Effect magnitude and estimation of whether subtest-level Flynn Effects are proportional to *g* loadings. Second, data collection ran over four decades, enabling us to test whether mean-level cognitive ability scores increased across generational cohorts (cohort effects). Three versions of the Wechsler Intelligence Scale for Children (WISC) were administered during the study, making it possible to estimate whether test re-standardization resulted in decreases in mean cognitive ability scores (test version effects). With this data structure, we could estimate both cohort and test version effects simultaneously within a single sample, thereby mitigating the limitations of each approach and increasing confidence in the robustness of any observed Flynn Effects. Third, children were followed prospectively, with some children taking multiple versions of the WISC over the course of their participation. This generated longitudinal data that can be leveraged to address limitations of cross-sectional Flynn Effect analyses.

Fitting multivariate models that pooled information from all available cohorts, subtests, and ages, we investigated whether Flynn Effect magnitude was consistent across cognitive domains, and whether subtest gains were associated with gains on general cognitive ability, while also boosting statistical power (Giangrande et al., 2019). We hypothesized that we would observe evidence of the Flynn Effect in two ways: 1) for a given age and test version, children who participated more recently in the LTS would have higher cognitive ability scores on average than previous cohorts; 2) for a given age and cohort, children who took newer WISC versions would score lower on average than children who took older versions. Based on the results of Giangrande et al. (2022) and previous findings, we expected to observe significant effects on FSIQ, VIQ, and PIQ, and predicted that Flynn Effects on PIQ (fluid intelligence) would be stronger than VIQ (crystallized intelligence). In analyses of Flynn Effects on individual subtests, we expected that the magnitude of the effect would fluctuate somewhat across subtests in a manner that was not necessarily proportional to subtest loadings on general cognitive ability, consistent with previous literature. However, we did not make specific hypotheses about our subtest-specific analyses due to a lack of consistency in previous research, so they should be considered exploratory.

## Methods

### Participants

We analyzed Louisville Twin Study data from 1187 individuals collected at ages 7, 8, 9, 12, and 15 years (3844 individual cognitive measurements; Table 1). All twins were from in and around Louisville, Kentucky in the United States. Data were collected between 1957 and 1999, with most children entering the study shortly after birth and followed prospectively until age 15. Age 12 data were not collected until 1984 (Davis et al., 2019), resulting in a smaller sample at that age. The sample represents the full socioeconomic status spectrum (Wilson, 1983) but is relatively racially and ethnically homogeneous. Approximately 90% of children were designated as White. Black individuals comprised nearly all the remaining 10% of the sample. These percentages do not reflect Louisville’s demographics at the time, underrepresenting Black individuals: Louisville was 82%, 76%, 71%, and 69% White in 1960, 1970, 1980, and 1990, respectively, and 18%, 24%, 28%, and 30% Black (United States Census Bureau, 2005). The sample was 52% female and of average IQ. 87% of participants were tested at two or more ages. Supplementary Table 1 summarizes longitudinal data coverage.

### Measures

We analyzed WISC, WISC-R, and WISC-III scores (Wechsler, 1949, 1974, 1991) that were age-scaled against national norms. Test version was coded as −1, 0, or 1 for WISC, WISC-R, and WISC-III, respectively. Both index scores (i.e., full-scale IQ, Performance IQ, Verbal IQ) and scores on the subtests that comprise those index scores were available. Six verbal IQ subtests (Information, Comprehension, Arithmetic, Similarities, Vocabulary, and Digit Span) and six performance IQ subtests (Picture Completion, Picture Arrangement, Block Design, Object Assembly, Coding, and Mazes) were administered. Very few original WISC Mazes scores were available because, in accordance with WISC administration guidelines, it was treated as an “alternate subtest” in the early days of the LTS, only to be used when a core performance subtest was invalid. We therefore omitted Mazes from all analyses, consistent with other subtest-level Flynn Effect studies (Kanaya & Ceci, 2011). In analyses focused on *g* (detailed below), we estimated general cognitive ability as a single latent factor comprising the 11 subtests. Examining the extent to which Flynn Effects were associated with gains in *g*, a single factor, is consistent with most of the existing literature on this topic, including early conceptualizations of the problem (e.g., Jensen, 1998) and previous empirical studies. However, beyond general cognitive ability (e.g., FSIQ), the WISC is known to consist of multiple subfactors (e.g., factors corresponding to VIQ and PIQ). Focusing our analysis on *g* therefore prioritized consistency with the previous literature at the potential expense of resolution in the cognitive factor structure.

For age, we used the earliest participants’ date of birth (DOB; in May, 1956) as an anchor. All other children’s DOBs were then defined as the number of days between their actual birthdays and the anchor DOB. To simplify interpretation, we rescaled DOB in decades by dividing each child’s DOB (days between actual DOB and the anchor) by 3650 days (i.e., 10 years).

### Procedure

Data preparation, calculation of descriptive statistics, and plotting was conducted in R (R Core Team, 2025). Code is freely available at https://github.com/egiangrande/Flynn-MIMIC. We fit all models in Mplus Version 8 (Muthén & Muthén, 1998/2017) using full information maximum likelihood to handle missing data. In all analyses, we corrected standard errors to account for the fact that twins came from the same family and therefore would be expected to have more similar scores than unrelated individuals. Our analysis proceeded in two stages. First, we estimated Flynn Effect magnitude across cognitive domains and subdomains by fitting a latent growth curve (LGC) model separately to each index and subtest score. Then, we tested the extent to which subtest level gains were or were not proportional to loadings on *g* by extending our LGC model into a multiple indicators, multiple causes (MIMIC) framework.

### Latent growth curve model on individual index and subtest scores

Our first model tested whether the Flynn Effect (in the form of both cohort and test version effects) influenced individual index and subtest scores using an LGC approach (Figure 1). We fit this model separately to each of the three cognitive ability index scores (FSIQ, VIQ, and PIQ) and the 11 subtest scores. LGC models characterize cognitive development using two latent factors. The first, *intercept* (I), pools information from all available time points to estimate a child’s performance at the initial measurement occasion. The second, *slope* (S), estimates the rate of change (i.e., growth) from that initial performance over time. We fixed all index/subtest loadings on the intercept to 1 and centered the growth model at the first time point by fixing the age 7 slope loading to 0. To facilitate interpretation, later-age loadings on the slope factor were weighted to account for different time gaps between measurement occasions (e.g., one year between ages 7 and 8; eight years between 7 and 15). Our results are conditional on this coding and may have differed somewhat had we used other weights. We regressed each index/subtest score on the score at the previous age to model autoregressive effects (Bollen & Curran, 2004).

To model cohort Flynn Effects, we regressed the intercept and slope factors on DOB. Based on our hypotheses, we expected that the regression of intercept on DOB would be positive, which would indicate that scores increased over the four decades of the study. Due to the lack of previous subdomain-level research on Flynn Effects on intraindividual change, we did not make specific hypotheses about Flynn Effects on slope. However, modeling intraindividual change in this way enabled us to control for age effects when estimating cohort effects (Schaie et al., 2005). To examine whether test re-standardization influenced scores, we regressed the observed index/subtest score at each age on test version at the concurrent age. We expected these regressions would be negative, indicating that scores decreased upon the introduction of a new WISC version. As mentioned above, test version was coded as −1, 0, and 1 for the WISC, WISC-R, and WISC-III, respectively). This coding specified a linear effect across successive test versions under the assumption that mean-level change difference between the WISC and WISC-R was equivalent to the WISC-R vs. WISC-III difference. We modeled test version effects on observed scores at each age (rather than on latent intercept and slope) because some children took more than one WISC version over the course of their participation in the LTS. Covariances were estimated among test version across ages and DOB.

### Longitudinal MIMIC model

The LGC model enabled us to test for the Flynn Effect in specific subtests. However, it did not allow us to determine whether gains on individual subtests were proportional to their loadings on *g;* i.e., whether subtest gains could be modeled in terms of Flynn Effects on g or independent of them. To address this question, we developed a longitudinal multiple indicators, multiple causes (MIMIC) model (LMM; Figure 2). Let k index subtests generally and j denote the focal subtest in a given model. At each age t, the 11 WISC subtest scores $Y_{itk}$ are modeled as reflective indicators of a latent factor $F_{it}$; with factor loadings $\lambda_{k}$. We constrained the factor loadings to be equivalent across ages.


(1) $${\text{Y}}_{itk}=\lambda_{k}*{\text{F}}_{it}+e_{itk},\;k\neq j$$

We fit a latent growth curve by estimating intercept and slope from the F factors. Thus, the basic structure of the LMM is analogous to our LGC model, except instead of estimating intercept and slope directly from *observed* IQ scores, we estimated them from general cognitive ability *factors*.


(2) $$F_{it}=I_{i}+a_{t}S_{i}+\gamma_{t}T_{it}+\varepsilon_{it}$$

Where $a_{t}=\{0,1,2,5,8\}$ for ages 7, 8, 9, 12, and 15, respectively. We estimated residual variances $\varepsilon_{it}$ for the latent factors, and estimated autoregressive paths among the latent factors (unpictured for simplicity). Test version effects $\gamma_{t}$ were estimated by regressing each latent cognitive ability factor $F_{it}$ on concurrent test version $T_{it}$: As in the LGC model, intercept and slope are regressed on DOB to estimate cohort effects ($\beta_{I}$ and $\beta_{S}$).


(3) $$I_{i}=\mu_{I}+\beta_{I}DOB_{i}+\zeta_{Ii}$$

(4) $$S_{i}=\mu_{S}+\beta_{S}DOB_{i}+\zeta_{Si}$$

Using the MIMIC approach, the observed score for a given subtest can then be modeled as a function of the general cognitive ability factor, cohort, and test version. Specifically, for a focal subtest j, we estimate direct effects of cohort and test version ($\alpha_{j}$ and $\rho_{j}$; respectively) on the observed scores for that subtest across ages while controlling for Flynn Effects on the general cognitive ability factor, as well as the strength of that subtest’s factor loading. Direct effect magnitudes were held constant across ages.


(5) $$Y_{itj}=\lambda_{j}F_{it}+\alpha_{j}DOB_{i}+\rho_{j}T_{it}+e_{itj}$$

Thus, the MIMIC approach enabled us to test whether the Flynn Effect can be modeled solely in terms of latent general cognitive ability, or whether individual subtests showed gains that were larger or smaller than what would be expected given their loadings on the general cognitive ability factor.

To get a baseline measure of Flynn Effects on general cognitive ability as estimated by the LMM, we first ran a base model that did not include any direct subtest effects. The factor structure of this base model assumes that the strength of the Flynn Effect is equivalent across subtests and proportional to each subtest’s loading on the general cognitive ability factor. We then fit the model including direct subtest effects 11 times, iterating through which WISC subtest was treated as the focal subtest. We analyzed whether direct Flynn Effects were significantly different from zero. Non-zero direct effects would indicate that the strength of the Flynn Effect on a given subtest is dis-proportionate to its loading on the general cognitive ability factor. Specifically, positive cohort direct effects or negative test version direct effects would suggest that subtests showed Flynn Effects that were above and beyond the Flynn Effect on general cognitive ability. In contrast, negative cohort direct effects or positive test version direct effects would indicate smaller Flynn Effects on subtests than what would be expected given the Flynn Effect on general cognitive ability. Because of the inconclusive results of previous studies, we did not make specific hypotheses about direct effects.

### Multiple comparisons

To account for multiple comparisons, we adjusted *p*-values using the Benjamini-Hochberg procedure (Benjamini & Hochberg, 1995), controlling the false discovery rate (FDR) at *q* = .05. We treated each model (LGC and LMM) as a separate family and then further divided tests by effect (cohort and test version). We then applied the Benjamini-Hochberg correction within each family (i.e., LGC cohort effects on intercept and slope, LGC test version effects on all subtests and all ages, LMM cohort effects on all subtests, LMM test version on all subtests).

### Model fit

We evaluated model fit using several indices: root mean square error of approximation (RMSEA), comparative fit index (CFI), Tucker-Lewis Index (TLI), standardized root mean square residual (SRMR) (see West et al., 2023 for a review). We compared fit statistics to heuristic cutoffs for each index: <0.06 for RMSEA, >0.95 for CFI and TLI, and <0.08 for SRMR (West et al., 2023). These cutoffs should not be considered firm guidelines, particularly for complex models that deviate from the confirmatory factor analysis models tested by Hu and Bentler (1999), such as the ones we fit here (McNeish, 2026; West et al., 2023).

## Results

### Latent growth curve model results

LGC model fit was excellent (Supplementary Table 2). LGC results (Table 2, Supplementary Table 3) supported our hypotheses. We observed significant Flynn Effects in the form of both positive cohort effects and negative test version effects for all WISC indices and most subtests (*p*s < 0.05; numerically, effects on index scores appeared larger than those on subtest scores because the former are scaled to a mean of 100 and the latter to a mean of 10). For the indices, cohort effects on intercept equaled a gain of approximately three IQ points per decade, with the numerically largest gains observed on VIQ (3.12, SE = 0.80) followed by FSIQ (3.05, SE = 0.68) and PIQ (2.93, SE = 0.75). Effects on slope were not significant, except for Comprehension (−0.15, SE = 0.04). Test version effects on the index scores were all significantly negative except for the effect on VIQ at age 12 (negative but not significant) and the PIQ effect at age 15 (did not survive FDR correction). Among significant test version effects on index scores, magnitude ranged from −2.94 (SE = 1.21, VIQ age 15) to −6.66 (SE = 0.84, PIQ age 9). These equal decreases of 5.88 and 13.32 IQ points, respectively, over the course of the study related to test re-standardization. At ages 7, 8, 9, and 12, test version effects were numerically strongest on PIQ followed by FSIQ and then VIQ. Test version effects remained stronger on FSIQ than VIQ at age 15, but were smallest for PIQ.

Eight out of 11 subtests showed at least one significant Flynn Effect estimate in the expected direction. Six subtests exhibited significant, positive cohort effects: Information (did not survive FDR correction), Comprehension, Similarities, Picture Arrangement, Block Design, and Coding. Among these, increases ranged from 0.40 (SE = 0.17; Information) to 1.04 (SE = 0.20; Coding) scaled score points per decade (or 2.00 and 5.20 IQ points per decade, respectively). Nearly all test version effects on subtests were negative (87%), most of those significantly so (60%). The only exceptions were Digit Span at ages 12 and 15 and Picture Completion at age 15, for which test version estimates were significantly positive (0.75 (SE = 0.32), 0.75 (SE = 0.33), and 0.89 (SE = 0.38), respectively, equaling gains of 7.50, 7.50, and 8.90 IQ points over the study), and Vocabulary, for which we did not observe any significant test version effects. Strength of test version effects among significant negative estimates ranged from −0.44 (SE = 0.24, Object Assembly age 7) to −1.77 (SE = 0.26, Similarities age 8) (or decreases of 4.40 and 17.70 IQ points, respectively, related to test re-standardization).

Of the six subtests that showed significant cohort effects, Information was the only subtest for which we did not also find a significant negative test version effect at any age. We found significantly negative test version effects for Arithmetic and Object Assembly (ages 7, 9, and 15 for Arithmetic; ages 7, 8, and 9 for Object Assembly) despite not observing significant cohort effects. Vocabulary was the only subtest for which we did not observe significant cohort or test version effects.

### Longitudinal MIMIC model results

Model fit was poorer for the LMM compared to the LGC (Supplementary Table 2). RMSEA = 0.065, slightly above the heuristic threshold of 0.06, but other fit statistics fell clearly below or above thresholds for other metrics: CFI and TLI = ~0.62, SRMR = ~0.12. This reduction in fit compared to the LGC model likely reflects the LMM’s single-factorial structure, added model complexity (which presents challenges when using traditional fit statistics), and that, although our LMM allowed us to test *g*-related questions directly on a subtest-by-subtest basis, it is not a comprehensive model of all subtests’ MIMIC effects (such a model would be prohibitively complex to fit). Results from the LMM base model (which did not include direct effects of cohort and test version on subtests) were consistent with LGC model results (Table 3, Supplementary Table 3) and in line with our hypotheses. Flynn Effects on general cognitive ability were clear: the cohort effect on cognitive ability intercept was significantly positive while test version effects on latent cognitive ability factors (F) were significantly negative across all ages (*p*s < 0.05). The magnitude of cohort-related gains and test version-related decreases were roughly commensurate with those observed in LGC analyses of index scores (gains of 3.10 IQ points per decade; mean decreases of 10.84 points due to test re-standardization over the study).

For most subtests, direct effect analyses revealed that the magnitude of the Flynn Effect was not proportional to strength of loading on the general cognitive ability factor (i.e., direct effects were significantly non-zero). Eight of 11 subtests exhibited either a significant cohort or test version direct effect, with both types of effects reaching significance for five of those (Information, Vocabulary, Picture Completion, Picture Arrangement, and Block Design, although the Block Design cohort effect did not survive correction for multiple comparisons). These deviations were small to moderate, overall. Absolute values of significant cohort direct effects ranged from 0.22 (SE = 0.10; Similarities) to 0.65 (SEs = 0.09 and 0.13 for Vocabulary and Coding, respectively), or 1.10 to 3.25 IQ points per decade, while absolute values of significant test version direct effects ranged from 0.37 (SE = 0.16, Digit Span) to 1.10 (SE = 0.13, Vocabulary), which equals changes in 3.70 to 11.00 IQ points over the course of two test re-standardizations. Direct effects were null on only Comprehension, Arithmetic, and Object Assembly.

Performance IQ subtests tended to exhibit Flynn Effects that were above and beyond those observed on latent cognitive ability (i.e., positive direct effects of DOB, negative direct effects of test version, or both). Direct cohort effects on Picture Completion, Picture Arrangement, Block Design, and Coding were significantly positive (0.23 (SE = 0.09), 0.64 (SE = 0.12), 0.21 (SE = 0.10), and 0.65 (SE = 0.13), respectively), although the Block Design effect did not survive FDR correction. Picture Completion, Picture Arrangement, and Block Design also showed significantly negative direct effects of test version (−0.52 (SE = 0.14), −1.03 (SE = 0.16), and −0.47 (0.14), respectively). We did not observe this pattern for any of the Verbal IQ subtests; they instead tended to display an opposite pattern of negative cohort direct effects (Information, Similarities, and Vocabulary: −0.38 (SE = 0.09), −0.22 (SE = 0.08), and −0.65 (SE = 0.09), respectively) or positive test version direct effects (Information, Vocabulary, and Digit Span: 0.92 (SE = 0.13), 1.10 (SE = 0.13), and 0.37 (SE = 0.16), respectively), which suggests that Flynn Effects on these subtests were smaller than what would be expected given the Flynn Effects on general cognitive ability.

## Discussion

This study examined the Flynn Effect at the level of general cognitive ability, domains, and subdomains across middle childhood and early adolescence using longitudinal models. Results provided clear evidence of the Flynn Effect in the Louisville Twin Study and highlight the utility of examining subtest-level gains. The size of intergenerational increases in mean cognitive ability fluctuated across domains and subdomains, which has important implications for how researchers and practitioners adjust for the Flynn Effect. Using a MIMIC-based approach, we found that subtest-level gains were not directly proportional to *g*-loadings.

### Substantial gains at the general, domain, and subdomain levels

For general cognitive ability, we observed substantial gains in mean general cognitive ability scores as increasing mean scores across generations (cohort effects) and sudden decreases in mean scores upon the introduction of a newer WISC version (test version effects) in LGC analyses of FSIQ and LMM analyses of a latent, general cognitive ability factor. Effect sizes were roughly consistent with Flynn’s original finding of three IQ points per decade (Flynn, 1984, 1987) and with hundreds of subsequent studies that have observed similar estimates—at least in data collected throughout the 1900s—in samples from around the world (Pietschnig & Voracek, 2015; Rodgers, 2023; Trahan et al., 2014; Wongupparaj et al., 2015, 2023). LGC analyses revealed that the presence and magnitude of the Flynn Effect fluctuated across cognitive domains. Among index scores, test version effects were numerically strongest in PIQ (fluid intelligence) followed by FSIQ (general intelligence) and then VIQ (crystallized intelligence) at four out of five ages (PIQ showed the smallest effect at age 15, but the effect on FSIQ was still larger than that on VIQ), consistent with our hypotheses. Previous studies have found the same pattern (Flynn Effects on fluid intelligence> FSIQ> crystallized intelligence; Pietschnig & Voracek, 2015). Interestingly, this pattern was mirrored in cohort effects on index score intercepts; the largest effect was on VIQ, followed by FSIQ and then PIQ. The reason for this is not entirely clear, although we should exercise caution when interpreting possible differences across index-level cohort effects given the size of the standard errors. The fact that the relative size of the Flynn Effect across cognitive domains differed by estimation approach speaks to the utility of modeling both test version and cohort effects, as we did here.

The presence and magnitude of the Flynn Effect also fluctuated across individual subtests. In LGC analyses, eight out of 11 subtests displayed at least one significant cohort or test version effect in the expected direction. However, even among subtests for which Flynn Effects were significant, the strength of the Flynn Effect varied considerably (cohort effects: increases of 2.00 and 5.00 IQ points for Information (nominally significant) and Coding, respectively; test version effects: decreases of 4.40 and 17.70 IQ points for Object Assembly age 7 and Similarities age 8, respectively). Unlike the index scores, neither significance nor magnitude followed a clear pattern along subdomain lines (e.g., PIQ subtests exhibiting different gains than VIQ subtests). Consistent with an emerging literature that examines the Flynn Effect across subdomains (Dworak et al., 2023; Oberleiter et al., 2024), these results suggest that the nature of the Flynn Effect is more nuanced than would be apparent by looking solely at index scores. Five of the eight subtests showed both cohort and test version effects, one showed only a cohort effect (Information), and two (Arithmetic and Object Assembly) showed only test version effects. As with the observed differences in cohort and test version gains in index scores, this could indicate that the strength of observed Flynn Effects on a given cognitive subdomain varies depending on the study design used (i.e., cohort vs. test version). In cases where we observed a significant cohort effect but at least one test version effect was not significant, sample size could have played a role: cohort effects on intercept were estimated by pooling available data from all five ages, maximizing sample size, while test version effects were estimated within-age and were likely less powerful.

LGC analyses yielded surprising findings for two subtests: test version effects were positive for Digit Span at ages 12 and 15 and for Picture Completion at age 15 (gains of 7.50, 7.50, and 8.90 IQ points, respectively). No other test version effects were significant for these subtests. If robust, these results would suggest that mean Digit Span and Picture Completion scores *increased* upon the introduction of a new WISC version. It could be that test re-standardization was insufficient for these subtests, failing to account for the Flynn Effect fully, or that changes in test content made the newer versions easier, though more research is needed to address this possibility.

Using longitudinal data to model the slope of cognitive ability in LGC and LMM analyses enabled us to control for age effects when measuring cohort effects, thereby yielding more stable Flynn Effect estimates (Dickinson & Hiscock, 2010; Schaie et al., 2005). Except for Comprehension, we did not observe any significant cohort effects on slope. It is likely that our use of age-scaled scores (rather than raw scores) impeded our ability to detect slope effects, should they exist. By definition, scaling scores results in means and standard deviations that remain constant across ages, which flattens intra-individual change in unstandardized cognitive ability across childhood.

### Fluid vs. crystallized gains

For decades, researchers have debated the relative strength of Flynn Effects on measures of fluid intelligence and crystallized intelligence (Pietschnig & Voracek, 2015). The Louisville Twin Study data structure offered a unique opportunity to examine this question at both the index and subtest levels. On the one hand, our results were consistent with previous evidence suggesting that intergenerational increases in fluid intelligence measures are larger than crystallized intelligence gains (e.g., Pietschnig & Voracek, 2015). In LGC analyses, we observed numerically larger test version effects on PIQ intercept than VIQ (which measure fluid and crystallized intelligence, respectively). PIQ subtests showed particularly strong gains given Flynn Effects on general cognitive ability, while VIQ subtest gains were smaller than expected. On the other hand, cohort effects on VIQ intercept were numerically (although modestly) larger than PIQ effects, and subtest-level LGC gains did not follow a discernable fluid vs. crystallized pattern. This ambiguity highlights the need for additional Flynn Effect studies that span cognitive domains and subdomains. Regardless of relative fluid vs. crystallized magnitude, our results provide clear evidence for Flynn Effects on both fluid and crystallized index and subtest scores.

### Subtest gains not strictly proportional to g-loadings

LMM results demonstrated that the magnitude of the Flynn Effect was not strictly proportional to general cognitive ability factor loading for most subtests. Consistent with most (Andrzejewski et al., 2024; Colom et al., 2023; Lazaridis et al., 2022; Must et al., 2003; Oberleiter et al., 2024; Pietschnig et al., 2023; Pietschnig & Voracek, 2015; Te Nijenhuis, 2013; Wicherts et al., 2004), but not all (Colom et al., 2001; Oberleiter et al., 2025), previous *g*-focused Flynn Effect studies, this finding suggests that subtest gains have not been directly proportional to gains in *g.* Our base LMM of Flynn Effects on latent general cognitive ability, which assumed equivalent effects across subtests, captured the Flynn Effect in our data reasonably well, uncovering cohort and test version effects. However, when we modeled direct effects of test version and cohort on each individual subtest, controlling for Flynn Effects on general cognitive ability, the strength of the Flynn Effect deviated modestly, yet significantly, from strength of the loading on the general cognitive ability factor for eight out of 11 subtests (at least one of the direct effects of test version and cohort was significant for Information, Similarities, Vocabulary, Digit Span, Picture Completion, Picture Arrangement, Block Design, and Coding). The base LMM underestimated Flynn Effects on performance subtests while overestimating effects on verbal subtests. This suggests that consistent with LGC test version effect results and with previous literature, Flynn Effects on performance/fluid intelligence measures were particularly strong.

These results are consistent with the theory that differential Flynn Effects across cognitive domains and subdomains may stem from generational increases in cognitive ability specialization (Oberleiter et al., 2024). As environmental enrichment improves and society modernizes, people are increasingly rewarded for specializing cognitively and able to do so. It becomes advantageous, for example, to complete training in a particular field and pursue a specialized career. This accelerated specialization leads to greater population-level cognitive ability differentiation. Although environmental improvements boost cognitive ability generally, resulting in the classic Flynn Effect, some (sub)domains are reinforced more than others, leading to variability in cognitive gains that are not fully proportional to *g* loadings.

Some (e.g., Jensen, 1998) might counter that the secular gains in cognitive ability we observed are “hollow” because subtest gains are not strictly proportional to loadings on general cognitive ability. We believe that this position is incorrect for two reasons. First, it rests on the misguided equating of g as a statistical construct with the psychological construct of intelligence (see Kovacs & Conway, 2019). While g may be a useful way of indexing intelligence and is interesting statistically, g is not intelligence per se. Other models of intelligence exist that are equally as plausible as the g theory and are fully compatible with the finding that Flynn Effects result in differential gains across subtests (e.g., mutualism; Van Der Maas et al., 2006). Thus, it is possible to say that cognitive ability has risen even if gains have not been strictly g -proportional. Second, IQ gains being driven by some subtests rising independent of their g loadings does not mean that those increases are unimportant. We strongly concur with Flynn and colleagues (Flynn et al., 2014) that labeling such gains as “hollow” makes no more sense than does dismissing cognitive deficits experienced due to iodine deficiency, prenatal cocaine exposure, fetal alcohol syndrome, or traumatic brain injury as insignificant. Just as those deficits are meaningful even though they do not track exactly with g, secular *gains* can be meaningful regardless of whether subtest changes are proportional to loadings on a general cognitive ability factor.

### Fit and factor structure

Analyzing g —a single-factor model of cognitive ability—added to a body of g -focused Flynn Effect studies that spans decades. However, single-factor models may not fully capture the Flynn Effect’s intricacies. Although WISC subtests share variance that can be attributed to a single, general cognitive ability factor, factor-analytic studies have also identified structure at the domain level (Cohen, 1959; Reynolds & Ford, 1994). Thus, fitting a single-factor model to WISC subtest scores may have contributed to the LMM fitting worse than the LGC model. Multifactorial MIMIC models might fit the data better, and would make it possible to test whether intergenerational gains on a specific subtest are proportional to gains on the corresponding cognitive domain (e.g., whether Similarities gains are proportional to gains on verbal intelligence). Based on our g results and the cognitive specialization hypothesis discussed above (Oberleiter et al., 2024), we would expect that subtest gains would not be directly proportional to domain-level factor loadings, but this remains an area for future research. In addition to improving model fit, moving beyond g toward more nuanced cognitive ability frameworks may help unlock a deeper understanding of the Flynn Effect’s locus and causes. Our analyses, along with previous studies, have revealed that Flynn Effect magnitude varies across cognitive (sub)domains and subtests, but more work is needed to clarify the relative magnitude of gains across components of cognitive ability and identify elements contributing to this heterogeneity.

## Implications and future directions

Our results have important implications for researchers studying cognitive development. Previous research demonstrated that the Flynn Effect can substantially bias analyses that include multiple generational cohorts or cognitive test versions. Our current findings build on a small, but growing literature (Dworak et al., 2023; Oberleiter et al., 2024) showing that there is more going on under the hood of the Flynn Effect than often realized. Sufficient correction for the Flynn Effect requires careful consideration of both generational cohort and test version, as well as the psychometric granularity of the planned analysis (i.e., composite vs. domain vs. subtest scores). A reevaluation of past cognitive studies that did not adequately correct for the Flynn Effect is warranted.

This study identified several exciting avenues for future Flynn Effect research. Our results underscore the utility of looking beyond g and analyzing cognitive ability gains at the domain and subtest level. In addition to offering a more detailed understanding of the Flynn Effect and how to correct for it, granular analyses can help narrow the search grid for specific environmental factors that boost cognitive ability, helping untangle a key question in developmental research. Further investigation of how differences in estimation approach—cohort or test version—can yield different effect sizes may help clarify our understanding of the Flynn Effect and discrepant results in the existing literature. The longitudinal models developed in this study overcome limitations of cross-sectional Flynn Effect analyses (see Dickinson & Hiscock, 2010 and Schaie et al., 2005 for discussion), boost power (Giangrande et al., 2019), and help situate the Flynn Effect in a developmental context.

## Strengths and limitations

Strengths of the present study include subtest-level WISC data, which enabled multivariate Flynn Effect analyses at both the common factor and individual subtest levels; simultaneous analysis of cohort and test version effects in a single sample, enabling comparison of the effect sizes obtained using each approach and mitigating each approach’s limitations; the ability to test for the Flynn Effect across a long time span (four decades) and three test versions; data from across middle childhood and early adolescence, an important developmental period for which cohort effects have been rarely documented; and a high proportion of participants with longitudinal data.

This study also faces several limitations. First, our results may not necessarily generalize beyond the LTS sample. Given that the most recent data were collected over 20 years ago using WISC versions that are outdated today, results do not speak to the Flynn Effect’s current status or its presence in other generational cohorts. A popular area of Flynn Effect research has examined whether cognitive ability gains have continued into the twenty first century. Some studies have concluded that the Flynn Effect has recently slowed or even reversed in developed countries (Dworak et al., 2023; Teasdale & Owen, 2005). Others have argued that while a dampening of the Flynn Effect is plausible, a turnaround is unlikely and may instead arise from methodological issues (Gonthier & Grégoire, 2022; Rodgers, 2023). In any case, LTS data collection ended in 1999, largely preceding the start of the supposed reversal (which most studies put sometime around the turn of the twenty first century) and we did not analyze temporal changes in Flynn Effect strength. Similarly, we did not test whether the strength of the Flynn Effect varies with age, which is an open question in the literature (see Pietschnig & Voracek, 2015; Trahan et al., 2014; Wongupparaj et al., 2023 for conflicting, meta-analytic results). We analyzed data across ages 7–15 years, and the extent to which our results may have differed had we studied other developmental periods is unclear. Further, our sample was not racially or ethnically representative with respect to the Louisville or United States population at the time, with the vast majority of participants designated as White.

Second, our results do not rule out the possibility that measurement artifacts could have influenced observed test version effects (Beaujean & Osterlind, 2008; Ceci & Kanaya, 2010; Gonthier & Grégoire, 2022; Kaufman, 2010; Wicherts et al., 2004). However, the cohort effects we observed provide independent evidence that cognitive ability scores did indeed rise and suggest that test version effects at least partially reflected Flynn Effect gains. Third, our use of scaled scores limited our ability to detect Flynn Effects on rate of change in cognitive ability over time (slope). Fourth, consistent with previous studies that have focused on *g*, we fit single-factor MIMIC models of cognitive ability. Fitting more complex, multi-factor models would better reflect the multifactorial structure of cognitive ability and therefore could improve our understanding of Flynn Effects across cognitive domains. Fifth, we assumed measurement invariance over age and cohort. Other recent *g*-focused Flynn Effect studies have made the same assumption (Oberleiter et al., 2025) and previous studies of measurement-invariant scores have observed Flynn Effects (Lazaridis et al., 2022; Oberleiter et al., 2024). However, we did not examine measurement invariance directly here, and it therefore remains possible that age and generational changes in the structure of latent cognitive ability affected our results. Similarly, we were unable to examine differential item functioning due to a lack of item-level measures in the archival LTS data. We also assumed equivalent direct effects across age. Sixth, although our LGC model fit the data well per traditional statistics, fit was poorer for the LMM. Modeling cognitive ability as a single factor was consistent with previous g -focused Flynn Effect studies, but the LMM did not fully capture cognitive ability’s multifactorial structure and therefore likely compromised fit. Further, traditional fit indices and cutoffs should be treated as approximate, but not firm thresholds for complex models such as the LMM (McNeish, 2026; West et al., 2023), and the observed discrepancy between RMSEA (which was slightly above the heuristic threshold of 0.06) and other global fit statistics does not identify particular sources of misspecification (Lai & Green, 2016). Emerging approaches such as Dynamic Fit Index cutoffs present opportunities to examine global model fit more robustly and appropriately for models such as the LMM (McNeish, 2026; West et al., 2023), but statistical packages for our particular use case have not been developed yet and we did not conduct such analyses ourselves here. Because our model directly tested our question of interest—the relative influence of the FE on subtests above and beyond the FE on g – we felt comfortable interpreting the LMM’s results cautiously, despite these fit limitations. Finally, the LMM estimated direct effects in separate, focal subtest-specific models, rather than jointly (i.e., a model that fit direct effects across subtests simultaneously). Although joint estimation would raise identification challenges, the present approach does not isolate subtest-specific direct effects while accounting for direct effects on other subtests.

## Conclusions

This study demonstrated that the magnitude of the Flynn Effect varied across cognitive subtests in a manner that did not directly align with each subtest’s loading on general cognitive ability $\left(g\right)$. These results are consistent with theories suggesting that the environmental influences driving the Flynn Effect have facilitated cognitive differentiation at a population level. Future studies integrating cognitive subdomain-level data, complementary Flynn Effect estimation methods (e.g., cohort and test version effects), and repeated measures may help unpack the Flynn Effect’s complexity, situating it in its historical, developmental, and cognitive context.

## Supplementary Material

Supp 1

Supplemental data for this article can be accessed online at https://doi.org/10.1080/00273171.2026.2707653.

## Figures and Tables

**Figure 1.**
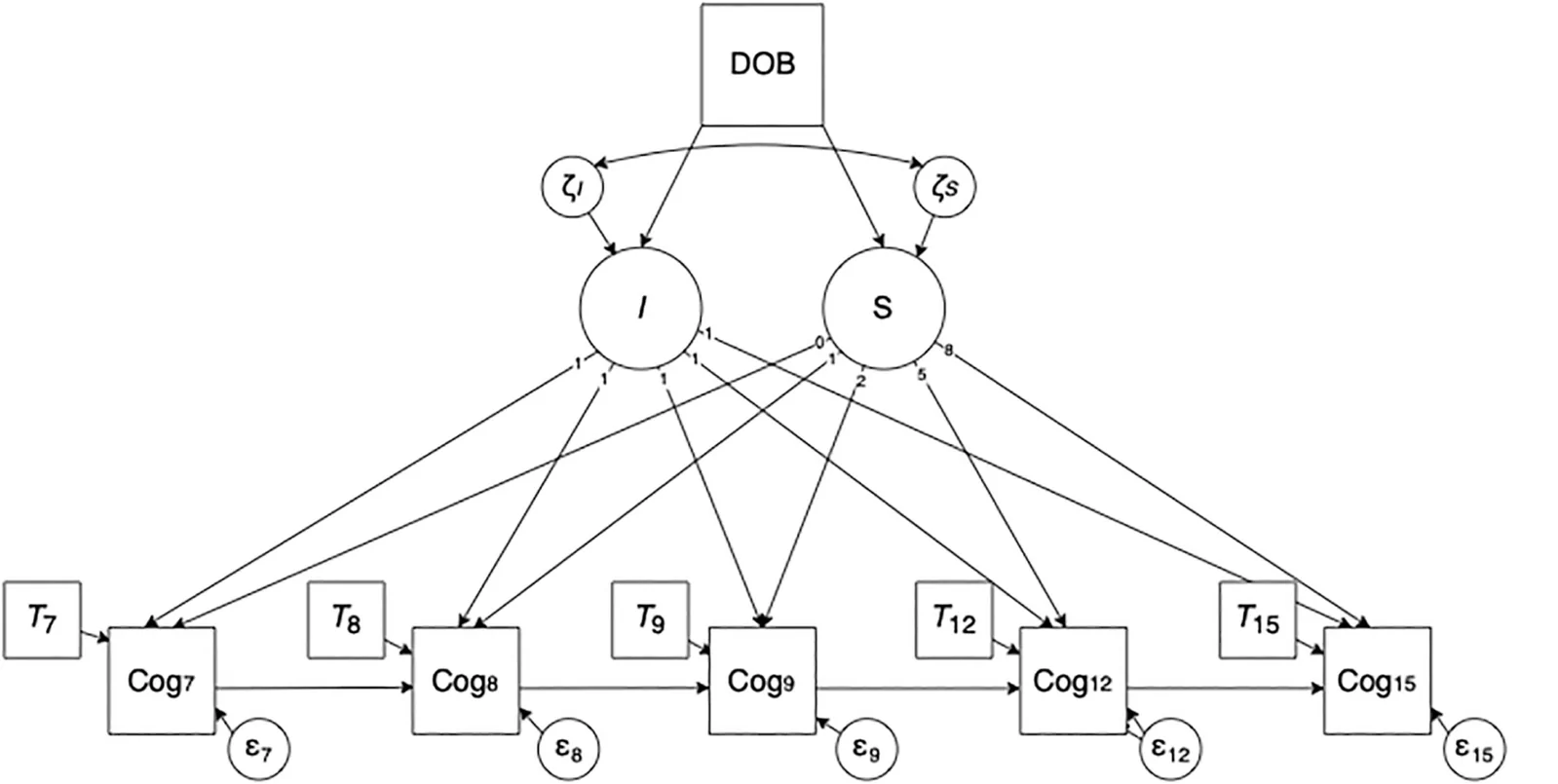
Latent growth curve model. *Note*. Cog: placeholder for the various cognitive ability index and subtest scores we analyzed. *I*: latent intercept factor. *S*: latent slope factor. ζ: LGC factor residual. ε: index/subtest score residual. DOB: date of birth. *T*: WISC test version coded as −1, 0, 1 for WISC, WISC-R, and WISC-III, respectively. Intercorrelations among DOB and T at each age were estimated but are not pictured here.

**Figure 2.**
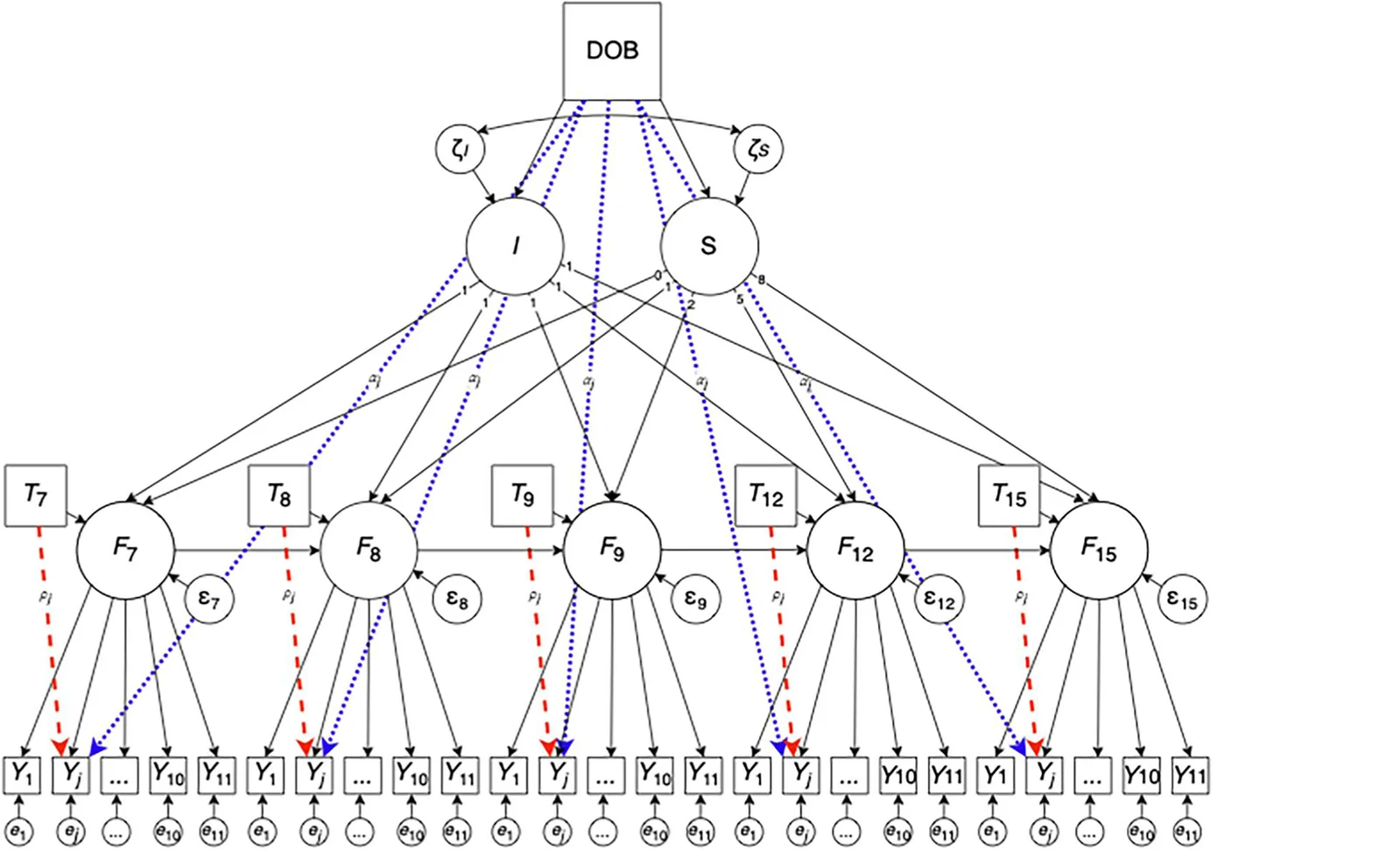
Longitudinal MIMIC model. *Note. I*: latent intercept factor. S: latent slope factor. ζ: LGC factor residual. DOB: date of birth. T: WISC test version coded as −1, 0, 1 for WISC, WISC-R, and WISC-III, respectively. F: latent cognitive ability factor. ε: index/subtest score residual. Y: WISC subtests, which are indicators of F. Only five subtests are depicted here for the sake of clarity; 11 subtests were analyzed. j: focal subtest, for which direct effects of cohort (α, dotted blue lines) and test version (ρ, dashed red lines) were estimated. e: subtest residual. Intercorrelations among DOB and T at each age were estimated but are not pictured here.

**Table 1.** Demographic and descriptive information.

Age (years)	n	% Female	% White/Black/ “Other”/ Multiracial	SES	FSIQ	% WISC/WISC-R/ WISC-III
7	966	54	89/9/1/1	47.83 (26.87)	98.16 (14.13)	23/56/21
8	1022	53	91/8/1/0	47.40 (26.35)	101.86 (14.02)	31/51/17
9	789	53	88/9/1/1	46.66 (27.12)	102.80 (14.45)	12/67/22
12	312	49	81/17/1/1	44.16 (28.84)	100.81 (14.39)	1/60/40
15	755	53	93/6/1/0	46.46 (26.29)	99.88 (14.06)	0/81/19
All Ages	1187	52	89/9/1/1	47.75 (26.64)	100.65 (14.27)	17/62/22

*Note*. SES: socioeconomic status. FSIQ: full scale IQ. Race data were missing for eight participants (four at age 8 and four at age 9). “Other” is the designation used in the archival [Louisville Twin Study] data; more detailed race information was unavailable. Some total percentages exceed 100 due to rounding.

**Table 2.** Latent growth curve model results.

Cog variable	I on DOB	S on DOB	Cog on T7	Cog on T8	Cog on T9	Cog on T12	Cog on T15
FSIQ	3.05 (0.68)*	−0.18 (0.12)	−5.23 (0.74)*	−5.14 (0.61)*	−5.93 (0.60)*	−4.05 (0.93)*	−3.52 (1.07)*
VIQ	3.12 (0.80)*	−0.24 (0.15)	−4.52 (0.96)*	−4.00 (0.78)*	−4.21 (0.73)*	−1.99 (1.12)	−2.94 (1.21)*
PIQ	2.93 (0.75)*	−0.29 (0.16)	−5.70 (0.92)*	−6.25 (0.84)*	−6.66 (0.84)*	−5.15 (0.99)*	−2.83 (1.38)†
Information	0.40 (0.17)†	−0.02 (0.04)	−0.33 (0.24)	−0.21 (0.19)	−0.23 (0.18)	0.19 (0.30)	0.29 (0.29)
Comprehension	1.00 (0.20)*	−0.15 (0.04)*	−1.15 (0.27)*	−0.81 (0.23)*	−1.28 (0.23)*	−0.77 (0.34)*	−1.05 (0.37)*
Arithmetic	0.33 (0.19)	−0.01 (0.04)	−1.00 (0.28)*	−0.43 (0.22)	−0.51 (0.22)*	−0.32 (0.32)	−0.80 (0.36)*
Similarities	0.72 (0.21)*	−0.04 (0.04)	−1.67 (0.30)*	−1.77 (0.26)*	−1.13 (0.21)*	−0.36 (0.30)	−0.32 (0.30)
Vocabulary	0.21 (0.18)	−0.02 (0.04)	−0.02 (0.24)	−0.24 (0.20)	−0.34 (0.22)	0.16 (0.34)	−0.53 (0.38)
Digit span	0.28 (0.18)	−0.02 (0.04)	−0.43 (0.24)	−0.12 (0.20)	0.03 (0.20)	0.75 (0.32)*	0.75 (0.33)*
Pic. Comp.	−0.15 (0.18)	−0.01 (0.04)	−0.32 (0.25)	−0.09 (0.22)	−0.15 (0.22)	−0.23 (0.39)	0.89 (0.38)*
Pic. Arrange.	0.89 (0.22)*	−0.11 (0.06)	−1.76 (0.31)*	−1.33 (0.26)*	−1.25 (0.26)*	−1.43 (0.31)*	−0.39 (0.53)
Block design	0.53 (0.16)*	−0.04 (0.04)	−1.07 (0.22)*	−1.05 (0.18)*	−1.23 (0.19)*	−1.11 (0.25)*	−1.15 (0.29)*
Obj. Assembly	−0.03 (0.17)	−0.07 (0.04)	−0.44 (0.24)*	−0.49 (0.20)*	−0.61 (0.20)*	−0.11 (0.29)	−0.41 (0.38)
Coding	1.04 (0.20)*	0.02 (0.04)	−0.96 (0.30)*	−1.41 (0.23)*	−1.82 (0.24)*	−0.65 (0.28)*	−1.23 (0.38)*

*Note*.

* *p* < .05.

† *p* < .05 but not significant after FDR correction. Cog: cognitive. I: intercept. S: slope. DOB: date of birth. T: test version. FSIQ: full scale IQ. VIQ: verbal IQ. PIQ: performance IQ. Pic.: picture. Comp.: completion. Arrange.: arrangement. Obj.: object. Subtest effects appear smaller than IQ effects because the former are scaled to a mean of 10 and the latter to a mean of 100.

**Table 3.** Longitudinal MIMIC model results.

Cog Variable	Cog on DOB	Cog on Test
I	0.31 (0.09)*	—
S	−0.02 (0.02)	—
F7	—	−0.61 (0.09)*
F8	—	−0.54 (0.08)*
F9	—	−0.62 (0.08)*
F12	—	−0.43 (0.12)*
F15	—	−0.51 (0.14)*
Information	−0.38 (0.09)*	0.92 (0.13)*
Comprehension	0.15 (0.09)	−0.16 (0.15)
Arithmetic	−0.09 (0.08)	0.09 (0.12)
Similarities	−0.22 (0.10)*	−0.23 (0.11)
Vocabulary	−0.65 (0.09)*	1.10 (0.13)*
Digit span	0.11 (0.12)	0.37 (0.16)*
Pic. Comp.	0.23 (0.09)*	−0.52 (0.14)*
Pic. Arrange.	0.64 (0.12)*	−1.03 (0.16)*
Block design	0.21 (0.10)†	−0.47 (0.14)*
Obj. Assembly	−0.18 (0.12)	−0.13 (0.16)
Coding	0.65 (0.13)*	−0.35 (0.19)

*Note*.

* *p* < .05.

† *p* < .05 but not significant after FDR correction. Cog: cognitive. DOB: date of birth. I: intercept. S: slope. F: latent cognitive ability factor. Pic.: picture. Comp.: completion. Arrange.: arrangement. Obj.: object. Cohort and test version effects on I, S, and F7-F15 are estimates from base longitudinal MIMIC model that did not include direct subtest effects.

## Data Availability

Code is freely available at https://github.com/egiangrande/Flynn-MIMIC. Louisville Twin Study data are not publicly accessible but are available upon reasonable request to the PIs.
