# Supplementary material for: To g or Not to g? A Cross-Domain, Subtest-Level Investigation of the Flynn Effect Across Ages 7–15 Years
Source: Multivariate Behav Res. Author manuscript; Available in PMC 2026 Nov 5. (PMC13738151; doi:10.1080/00273171.2026.2707653)
Supplement: Supplementary file 1 [file NIHMS2207189-supplement-Supp_1.docx]

**Supplementary Table 1**

*Longitudinal Data Coverage*

*Note*. Diagonal values indicate the proportion of the total sample that had cognitive data at each age. Off-diagonal values represent the proportion of the total sample available to calculate a covariance between cognitive measures at two ages.

**Supplementary Table 2**

*Model Fit Statistics*

Cog: cognitive. FSIQ: full scale IQ. VIQ: verbal IQ. PIQ: performance IQ. Pic.: picture. Comp.: completion. Arrange: arrangement.

**Supplementary Table 3**

*Autoregressive Effect Results*

*Note*. **p* < .05. LGC: latent growth curve. LMM: longitudinal MIMIC model. Cog: cognitive. I: intercept. S: slope. FSIQ: full scale IQ. VIQ: verbal IQ. PIQ: performance IQ. Pic.: picture. Comp.: completion. Arrange: arrangement.
